# Atorvastatin rescues hyperhomocysteinemia-induced cognitive deficits and neuroinflammatory gene changes

**DOI:** 10.1186/s12974-023-02883-x

**Published:** 2023-09-01

**Authors:** Erica M. Weekman, Sherika N. Johnson, Colin B. Rogers, Tiffany L. Sudduth, Kevin Xie, Qi Qiao, David W. Fardo, Teodoro Bottiglieri, Donna M. Wilcock

**Affiliations:** 1 Sanders-Brown Center on Aging, University of Kentucky, Lexington, KY 40536 USA; 2 Present Address: Stark Neurosciences Research Institute, Indiana University School of Medicine, Indianapolis, IN 46202 USA; 3 Center of Metabolomics, Institute of Metabolic Disease, Baylor Scott and White Research Institute, Dallas, TX 75204 USA

**Keywords:** Hyperhomocysteinemia, Vascular contributions to cognitive impairment and dementia, Atorvastatin, Neuroinflammation, Microglia, Microhemorrhages, Astrocyte end-feet, Cholesterol

## Abstract

**Background:**

Epidemiological data suggests statins could reduce the risk of dementia, and more specifically, Alzheimer’s disease (AD). Pre-clinical data suggests statins reduce the risk of dementia through their pleiotropic effects rather than their cholesterol lowering effects. While AD is a leading cause of dementia, it is frequently found co-morbidly with cerebral small vessel disease and other vascular contributions to cognitive impairment and dementia (VCID), which are another leading cause of dementia. In this study, we determined if atorvastatin ameliorated hyperhomocysteinemia (HHcy)-induced VCID.

**Methods:**

Wild-type (C57Bl6/J) mice were placed on a diet to induce HHcy or a control diet each with or without atorvastatin for 14 weeks. Mice underwent novel object recognition testing before tissue collection. Plasma total cholesterol and total homocysteine as well as related metabolites were measured. Using qPCR and NanoString technology, we profiled glial cell-associated gene expression changes. Finally, microglial morphology, astrocyte end feet, and microhemorrhages were analyzed using histological methods.

**Results:**

Atorvastatin treatment of HHcy in mice led to no changes in total cholesterol but decreases in total homocysteine in plasma. While HHcy decreased expression of many glial genes, atorvastatin rescued these gene changes, which mostly occurred in oligodendrocytes and microglia. Microglia in HHcy mice with atorvastatin were trending towards fewer processes compared to control with atorvastatin, but there were no atorvastatin effects on astrocyte end feet. While atorvastatin treatment was trending towards increasing the area of microhemorrhages in HHcy mice in the frontal cortex, it only slightly (non-significantly) reduced the number of microhemorrhages. Finally, atorvastatin treatment in HHcy mice led to improved cognition on the novel object recognition task.

**Conclusions:**

These data suggest that atorvastatin rescued cognitive changes induced by HHcy most likely through lowering plasma total homocysteine and rescuing gene expression changes rather than impacts on vascular integrity or microglial changes.

**Supplementary Information:**

The online version contains supplementary material available at 10.1186/s12974-023-02883-x.

## Background

Alzheimer’s disease (AD) and vascular contributions to cognitive impairment and dementia (VCID) are two leading causes of dementia and are frequently found co-morbid with the other. Epidemiologic data suggests that statins could reduce the risk of AD and dementia [1–3]. One retrospective cohort study in the United States reported that patients who used statins had a lower incidence of AD, dementia, Parkinson’s disease, and amyotrophic lateral sclerosis [4]. In another study looking at the dose response relationship between statins and incidence of AD, the authors found that persistent and adherent statin use was associated with a decreased risk of AD [5]. While there is evidence to support statins decrease the risk of dementia, there is also conflicting data suggesting statins either provide no benefit or in fact increase risk of dementia. One study looking at Korean adults over the age of 60, found that less persistent statin use was associated with increased risk of AD [5]. Several other studies and meta-analyses report no effects of statins on dementia risk [6–8]. While clinical trials are required to determine the efficacy, if any, of statins to reduce the risk of dementia, there have already been pre-clinical studies investigating the mechanism behind their possible role in reducing risk. Although their cholesterol lowering ability is beneficial and can help lower Aβ levels [9], the off-target effects of statins may play a bigger role in reducing the risk of dementia. Statins have been shown to have anti-inflammatory effects, with one study showing atorvastatin treatment caused a switch of microglia phenotype from pro- to anti-inflammatory after a traumatic brain injury [10]. In aged beagles, atorvastatin was also shown to reduce brain oxidative stress [11]. While the majority of research has focused on statins reducing the risk of dementia in general, the mechanism of statins reducing AD pathologies, or the benefits of statins through lowering cholesterol, we were interested in the ability of statins to reduce the risk of VCID through their anti-inflammatory effects.

One risk factor for VCID is hyperhomocysteinemia (HHcy), which occurs when plasma levels of homocysteine are elevated above 15 µmol/L. The most common cause of HHcy in the elderly is malabsorption of B vitamins, which are key co-factors required for the metabolism of homocysteine to and from methionine and for homocysteine conversion to cysteine [12–14]. There have been several studies investigating the mechanisms for how HHcy induces VCID. In our model of HHcy, induced via a diet deficient in B vitamins and enriched in methionine, one of the first pathologies we see is an increase in pro-inflammatory cytokines with an increase in microglia staining that eventually leads to loss of astrocyte end-feet, microhemorrhages and cognitive impairment [15, 16]. Since pre-clinical data for statins suggests an anti-inflammatory effect, we were interested in determining if statins would be effective in preventing the vascular pathology seen in our HHcy mouse model, and, therefore, would be a potential therapeutic treatment in the clinic.

In this study, we placed C57Bl6/J mice on a diet to induce HHcy or a control diet with or without atorvastatin for 14 weeks. We measured plasma total cholesterol and total homocysteine (tHcy) levels as well as hippocampal gene expression changes. We also identified changes in microglia and astrocyte end feet using immunohistochemistry. Finally, we measured microhemorrhages and cognitive changes using the novel object recognition task. Ultimately, atorvastatin rescued gene expression and cognitive changes induced by HHcy while having limited effects on microglial process length and number, did not alter astrocyte end feet and was only trending towards increased microhemorrhage area in the frontal cortex.

## Methods

### Animals

Fifty-four male and female C57Bl6/J mice were purchased from Jackson Laboratory and aged to 6 months before being placed on a diet deficient in vitamins B6, B9 and B12 and enriched in methionine (HHcy diet; n = 8F, 7M; TD.130867, Envigo, Indianapolis, IN) or a diet with normal levels of B vitamins and methionine (control diet; n = 6F, 6F; TD.01636, Envigo) with or without Atorvastatin (HHcy + ATO; n = 8F, 7M; TD.200023; control + ATO; n = 5F, 7M; TD.200022, Envigo) for 14 weeks. Atorvastatin calcium was obtained from Sigma (cat no: PHR1422, St. Louis, MO) and 100 ppm were added to each diet so that the mice were dosed with 20 mg/kg/day, which, based on the body surface area normalization method, equates to an intermediate dosage used in humans [17–19]. This study was approved by the University of Kentucky Institutional Animal Care and Use Committee and conformed to the National Institutes of Health Guide for the Care and Use of Animals in Research.

### Novel object recognition

Three days before euthanasia, animals were tested on the novel object recognition task. The testing apparatus consisted of a 40 cm × 40 cm × 40 cm open field box with four chambers set up and recorded from simultaneously. Mice were allowed to acclimate to the room for 30 min before testing began each day. On day one, mice were placed in the center of the container and were allowed to freely explore for 15 min. On day two, for the first trial, two identical objects were placed in opposite corners of the container. Each mouse was placed in the center of the container and allowed to explore freely for 5 min. Four hours later, one object was replaced with a novel object and the mouse was again placed in the center of the container and allowed to explore freely for 5 min. Interaction with the object was defined as the time the nose of the mouse was within 3 cm and facing the object. Time that animals spent standing on the object was excluded. Animals that spent less than 10 s total with either object were excluded from analysis. The percent time with the novel object was calculated as:

$$\% Time\, with \,Novel \,Object=(Time \,with \,Novel \,Object\div Time\, with \,Both \,Objects)\times 100$$

  

### Tissue collection and processing

Following a lethal injection of Euthanasia-III, blood was collected retro-orbitally into EDTA tubes, spun at 4 °C for 15 min at 1000*g*, plasma collected and stored at − 80 °C. Mice were perfused with 20 mL normal saline, brains were rapidly removed and bisected down the midsagittal plane. The right side was dissected into frontal and posterior cortex, hippocampus, striatum, thalamus, cerebellum and rest of brain and flash frozen in liquid nitrogen and stored at − 80 °C. The left side was fixed in 4% paraformaldehyde for 24 h for histology and then passed through 10%, 20% and 30% sucrose gradients prior to sectioning. On a sliding microtome, 25 µm frozen horizonal sections were collected and stored in 1xDPBS with sodium azide at 4 °C.

### Cholesterol assay

Mice underwent a blood draw every 4 weeks on diet. Blood was collected in EDTA tubes and spun at 4 °C for 15 min at 1000*g*, plasma was collected and stored at − 80 °C. Using a commercial kit, total cholesterol levels were measured on plasma taken from mice at 12 weeks on diet (Cholesterol Liquid Reagent Set and Cholesterol Standard, Pointe Scientific, Canton, MI). Briefly, 100 µL of cholesterol standards and sample (plasma samples diluted at 1:20 in distilled water) were added to a 96 well plate. 100 µL of the Cholesterol Liquid Reagent was added to each well and then incubated at 37 °C for 45 min and then absorbance at 500 nm was measured (Synergy HTOX Multimode Reader, Aligent, Santa Clara, CA).

### tHcy and metabolites

tHcy, methionine, S-adenosyl methionine (SAM), S-adenosyl homocysteine (SAH), cystathionine, betaine and choline were measured on plasma obtained from mice at 12 weeks on diet as previously described using the liquid chromatography-mass spectrometry method [20].

### RNA and NanoString

RNA was extracted from the right hippocampus using the E.Z.N.A. total RNA kit II (Omega-Bio-Tek, Norcross, GA) according to the manufacturer’s instructions. RNA quality and quantity was analyzed using the Aligent 2100 Bionanalyzer, located in the Genomics Core at the University of Kentucky. Normalized samples (10 ng/µL) were run on the NanoString Technologies nCounter Mouse Glial Profiling Panel, also located in the Genomics Core (NanoString, Seattle, WA).

NanoString gene expression changes were confirmed using qPCR. Using the High-Capacity cDNA kit, 100 ng RNA was reverse transcribed to cDNA according to the manufacturer’s instructions (Life Technologies, Carlsbad, CA). qPCR was performed as previously described [20]. All genes were normalized to 18s rRNA and the −ΔΔCt method was used for analysis.

### Immunohistochemistry and histology

Eight sections spaced 600 µm apart were used for immunohistochemistry. Immunohistochemistry was performed as previously described for ionized calcium binding adaptor molecule 1 (IBA-1, primary 1:1000, Wako, Osaka, Japan; rabbit secondary 1:3000), aquaporin 4 (AQP4, primary 1:1000, MyBioSource, San Diego, CA; rabbit secondary 1:5000), and dystrophin 71 (DP71, primary 1:1000, Abcam, Cambridge, UK; rabbit secondary, 1:5000) on an n = 4 or 5/group [20]. Stained sections were mounted, allowed to air dry overnight and then coverslipped in DPX (Electron Microscopy Sciences, Hatfield, PA). Slides were scanned using Nikon’s Bio-Pipeline and analyzed on Nikon Elements AR image analysis system (Nikon Instruments, Melville, NY). For AQP4 and DP71 vessel analysis, using the Nikon GA3 analysis, a threshold was set to determine positive stained area. From this, a binary area of positive stain was created, and each object/vessel was identified. The length and area of each vessel was calculated, then averaged over the frontal cortex and hippocampus.

Eight sections spaced 600 µm apart were stained using the Prussian blue protocol for microhemorrhages as previously described [20]. For analysis of microhemorrhages, using Indica Lab’s HALO software, a threshold was set to identify positive blue stain in the frontal cortex and hippocampus. Positive blue stain that was within two cell lengths from a vessel were included in the analysis, while ones that were not next to a vessel were excluded. Multiple positive blue stain along one vessel was counted as one microhemorrhage. The number of microhemorrhages were averaged per section and used for final analysis.

### NanoString analysis

Gene expression for all endogenous genes was investigated using multivariable linear regression models. For each regression model, the outcome was taken to be the log2 gene expression values for a particular gene and the predictors were taken to be the log2 of the sum of all positive control gene counts (to normalize the expression data), and an indicator for the appropriate comparisons made for the desired volcano plots. The overall effects of the comparisons’ p-values were converted to q-values to account for the large number of comparisons that were being made. There were not any statistically significant gene differences following multiple comparisons adjustment. All tests were two-sided. Observations with missing values were excluded on an analysis-by-analysis basis. All analyses were done in R programming language, version 4.0.4 (R Foundation for Statistical Computing, Vienna, Austria).

### Data analysis

All remaining data are represented as mean ± SEM. Statistical analysis was performed using JMP. Outliers were removed using the repeated Grubb’s test. Data were tested for normality. When data were normally distributed, a Tukey HSD was used to determine between group differences and control for multiple comparisons. When data was not normally distributed, a Steel–Dwass test was used to determine between group differences and control for multiple comparisons. Although all group comparisons were analyzed, only differences between control vs HHcy, control + ATO vs HHcy + ATO and HHcy vs HHcy + ATO were included in the figures except for the week 14 weight and the *ERBB3* data. For the *ERBB3* data, which was not normally distributed, a Steel test with one control, the control group, was performed since the NanoString data showed that *ERBB3* was downregulated in all groups compared to the control group. For the remaining qPCR data, a t-test or Wilcoxon test was used to compare the two groups as there was only one comparison.

## Results

**Fig. 1:**
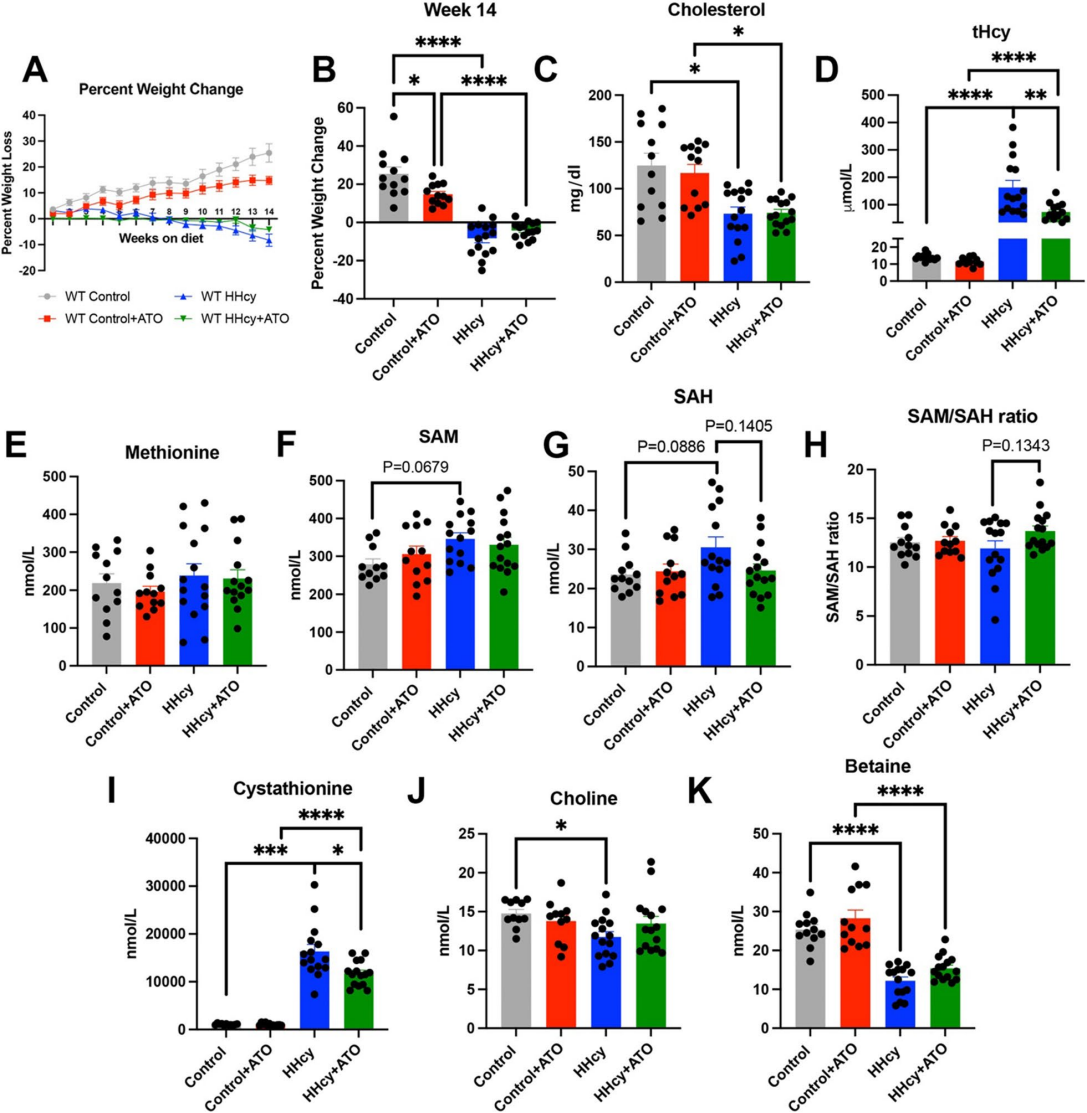
Atorvastatin altered the homocysteine pathway but not total cholesterol levels. Percent weight change over the course of the diet (**A**), and the final weight change at week 14 (**B**) are shown. Total cholesterol (**C**), plasma total homocysteine (**D**), methionine (**E**), S-adenosyl methionine (SAM) (**F**), S-adenosyl homocysteine (SAH) (**G**), SAM/SAH ratio (**H**), cystathionine (**I**), choline (**J**), and betaine (**K**) levels are shown. * indicates P < 0.05, ** indicates P < 0.01, *** indicates P < 0.001, **** indicates P < 0.0001

Weekly weights were taken for each mouse while on diet and the percent weight loss was calculated for each animal (Fig. 1A). While the mice on control diet continued to gain weight over the course of the study, the mice on control + ATO also gained weight, but not to the extent of the control mice (Fig. 1B). The mice on HHcy diet and HHcy + ATO diet lost weight compared to their control groups, but atorvastatin treatment did not rescue this weight loss by week 14 (Fig. 1B). Although we did not test for motor deficits or anxiety, we did not notice any general health problems in any groups of mice.

Plasma taken 3 months post diet induction was used to test total cholesterol and total homocysteine levels as well as related homocysteine metabolites. Mice on the HHcy diets (regardless of atorvastatin inclusion) had significantly lower total cholesterol levels compared to their control diets, but there was no effect of atorvastatin on cholesterol (Fig. 1C). Atorvastatin significantly lowered plasma total homocysteine in the HHcy diet group but was still within a moderate HHcy range (HHcy: 162.8 µmol/L, HHcy + ATO:73.1 µmol/L; 55% decrease in tHcy) (Fig. 1D). There were no significant changes in methionine, but the HHcy diet was trending towards increased SAM levels compared to controls (control: 279 nmol/L, HHcy: 346.1 nmol/L; 24% increase in SAM), with no changes due to atorvastatin (Fig. 1E, F). SAH levels were trending towards an increase in the HHcy group compared to control (control: 23.6 nmol/L, HHcy: 30.5 nmol/L; 29% increase in SAH), while atorvastatin was trending towards lowered SAH levels compared to the HHcy group (HHcy: 30.5 nmol/L, HHcy + ATO: 24.6 nmol/L; 19% decrease in SAH) (Fig. 1G). Due to the subtle changes in SAM and SAH levels, the mice on the HHcy with atorvastatin diet were also trending towards an elevated SAM/SAH ratios compared to the HHcy diet mice (HHcy: 11.9, HHcy + ATO: 13.7; 15% increase in SAM/SAH ratio) (Fig. 1H). The HHcy diet also increased cystathionine levels compared to both control groups (control: 1074 nmol/L, HHcy: 16344 nmol/L; 1421% increase in cystathionine; control + ATO: 1043.4 nmol/L, HHcy + ATO: 11736.7 nmol/L; 1024% increase in cystathionine) with a significant decrease due to atorvastatin in the HHcy group (Fig. 1I). HHcy significantly lowered choline levels compared to its control (control: 14.8 nmol/L, HHcy: 11.7 nmol/L; 20% decrease in choline), while atorvastatin did not (control + ATO: 13.8 nmol/L, HHcy + ATO: 13.5 nmol/L; 2% decrease in choline) (Fig. 1J). Atorvastatin did not rescue changes in betaine (control: 25.4 nmol/L, HHcy: 12.2 nmol/L; 52% decrease in betaine; control + ATO: 28.3 nmol/L, HHcy + ATO: 15.4 nmol/L; 45% decrease in betaine) (Fig. 1J, K).

**Fig. 2:**
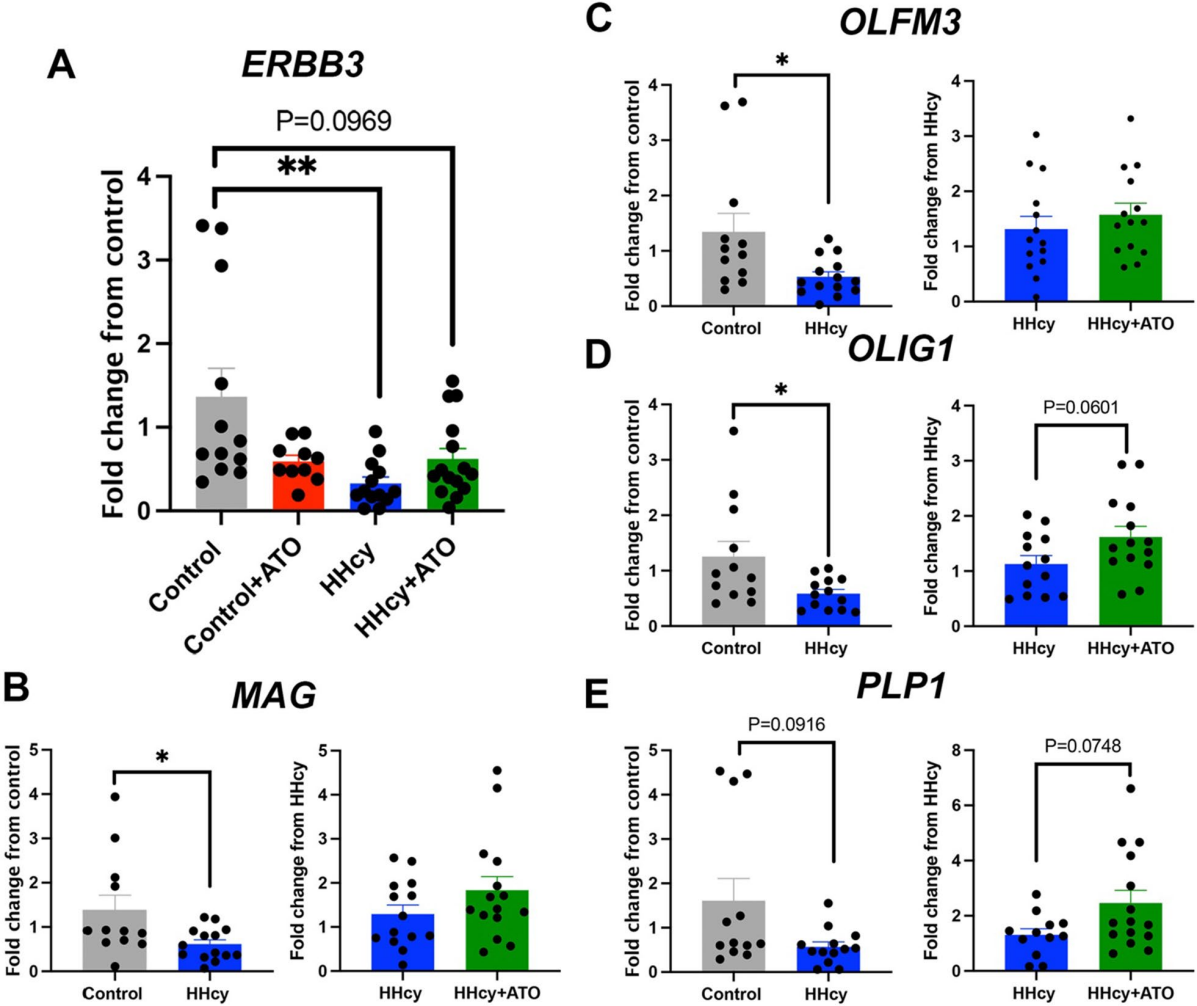
Hyperhomocysteinemia-induced downregulation of genes was rescued by atorvastatin. *ERBB3* (**A**), *MAG* (**B**), *OLMF3* (**C**), *OLIG1* (**D**) and *PLP1* (**E**) gene expression levels compared to mice on control diet or mice on the HHcy diet. * indicates P < 0.05

To determine glial gene expression changes associated with atorvastatin treatment, we performed NanoString’s Glial Profiling Panel on hippocampal RNA and confirmed with qPCR. While no genes passed false discovery rate (FDR) correction, there were trends in gene expression changes due to diet and treatment that were confirmed with qPCR. Receptor tyrosine-protein kinase erbB-3 (*ERBB3*), an activator of the MAPK and PI3K/Akt pathways, was significantly downregulated in the HHcy mice compared to control mice and was trending towards a decrease in the HHcy plus atorvastatin group (Fig. 2A). When compared to the control diet, HHcy significantly induced down regulation of myelin associated glycoprotein (*MAG*), olfactomedin 3 (*OLFM3*), and oligodendrocyte transcription factor 1 (*OLIG1*), and there was a trending decrease in proteolipid 1 (*PLP1*), most of which are related to oligodendrocytes (Fig. 2B–E). Atorvastatin with HHcy was trending towards increases in *PLP1* and *OLIG1* when compared to HHcy alone (Fig. 2D, E). The remaining NanoString gene expression changes with significant P values can be found in Additional file 1: Tables S1–S4.

**Fig. 3:**
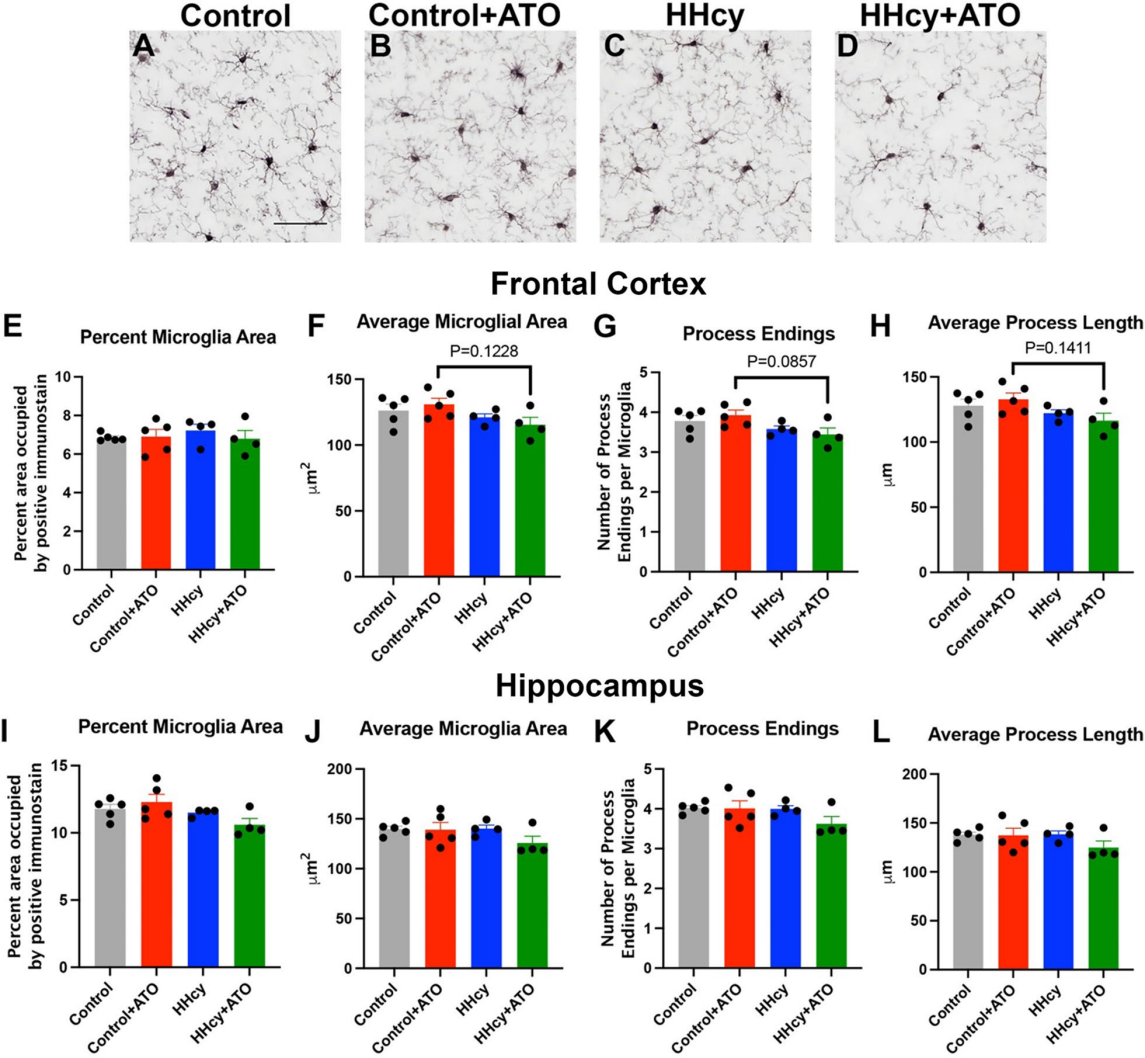
Hyperhomocysteinemia with atorvastatin leads to limited microglial changes  in the frontal cortex. Representative images of IBA-1 staining in the frontal cortex of mice on control (**A**), control + ATO (**B**), HHcy (**C**), and HHcy + ATO diet (**D**). Scale bar in A = 50 µm and is for images **A**–**D**. Percent microglia area (**E**), average microglia area (**F**), process endings (**G**) and average process length (**H**) in the frontal cortex are shown. Percent microglia area (**I**), average microglia area (**J**), process endings (**K**) and average process length (**L**) in the hippocampus are shown

While atorvastatin was trending towards glial gene expression changes, we were also interested in microglia morphology and staining changes. Immunohistochemistry for all microglia using IBA-1 showed no significant changes in percent area in the frontal cortex or hippocampus (Fig. 3A–E, I). The microglial area, average number of process endings per microglia, and average process length were trending towards a decrease in the HHcy with atorvastatin mice compared to control with atorvastatin in the frontal cortex, but not in the hippocampus, suggesting possible different regional responses to atorvastatin (Fig. 3E–L).

**Fig. 4:**
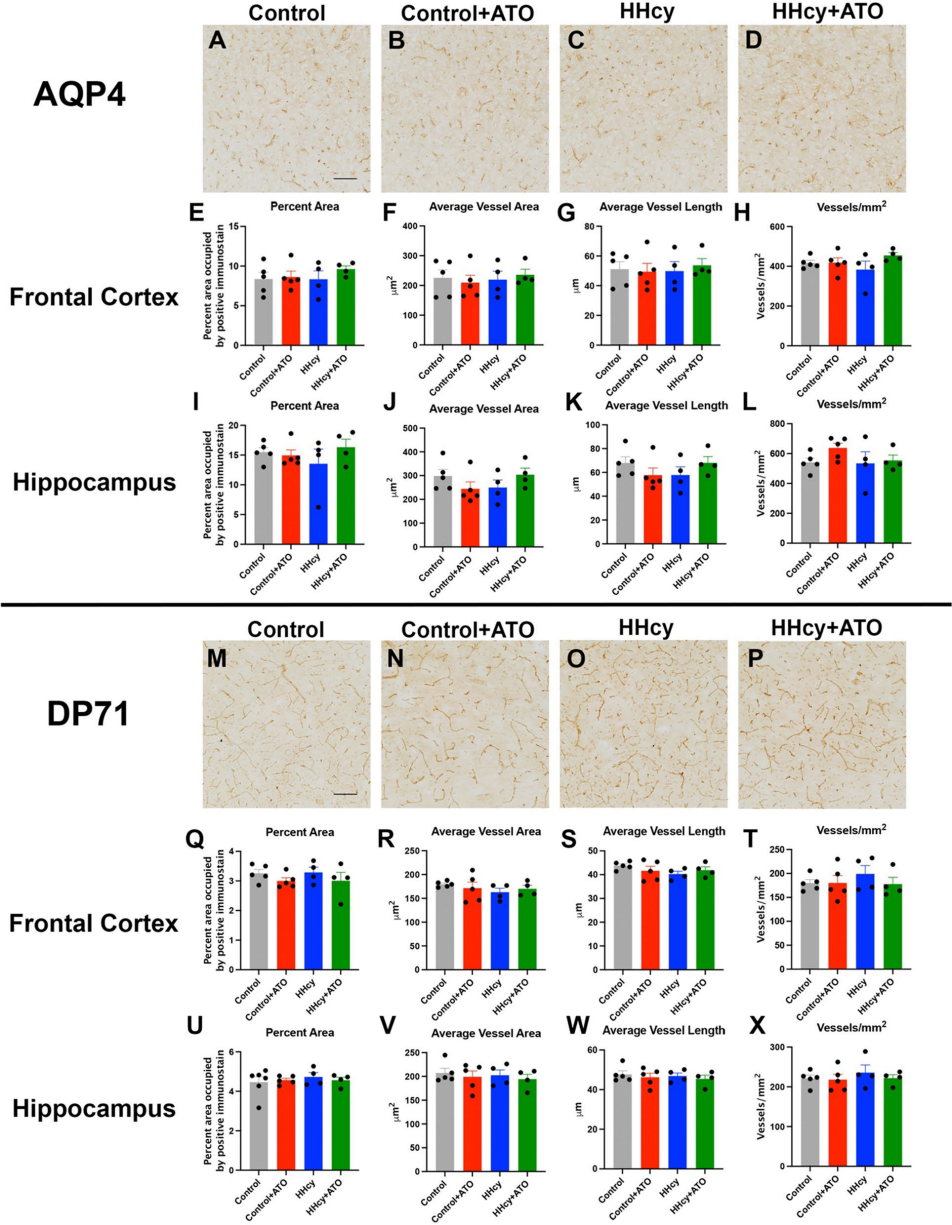
Atorvastatin does not alter astrocyte end-feet. Representative images of AQP4 staining in the frontal cortex of mice on control (**A**), control + ATO (**B**), HHcy (**C**), and HHcy + ATO diet (**D**). Percent area (**E**), average vessel area (**F**), average vessel length (**G**) and vessels/mm^2^ (**H**) in the frontal cortex are shown for AQP4. Percent area (**I**), average vessel area (**J**), average vessel length (**K**) and vessels/mm^2^ (**L**) in the hippocampus are shown for AQP4. Representative images of DP71 staining in the frontal cortex of mice on control (**M**), control + ATO (**N**), HHcy (**O**), and HHcy + ATO diet (**P**). Scale bar in A and M = 100 µm; scale bar in **A** is for images **A**–**D** and scale bar in **M** is for images **M**–**P**. Percent area (**Q**), average vessel area (**R**), average vessel length (**S**) and vessels/mm^2^ (**T**) in the frontal cortex are shown for DP71. Percent area (**U**), average vessel area (**V**), average vessel length (**W**) and vessels/mm^2^ (**X**) in the hippocampus are shown for DP71

In addition to microglia, we also investigated changes in astrocyte end-feet using AQP4 and DP71 immunohistochemistry. AQP4 is a water channel that is localized to the astrocyte end-foot while DP71 is part of an anchoring complex with dystroglycan and actin to help anchor the astrocyte end-foot to the basement membrane. We were interested in measuring both as one (AQP4) is related to the function of astrocyte end-feet while the other is a structural component (DP71). There were no significant changes in AQP4 in the frontal cortex or hippocampus (Fig. 4A–L). Similarly, there were no significant differences in DP71 in the frontal cortex or hippocampus (Fig. 4M–X).

**Fig. 5:**
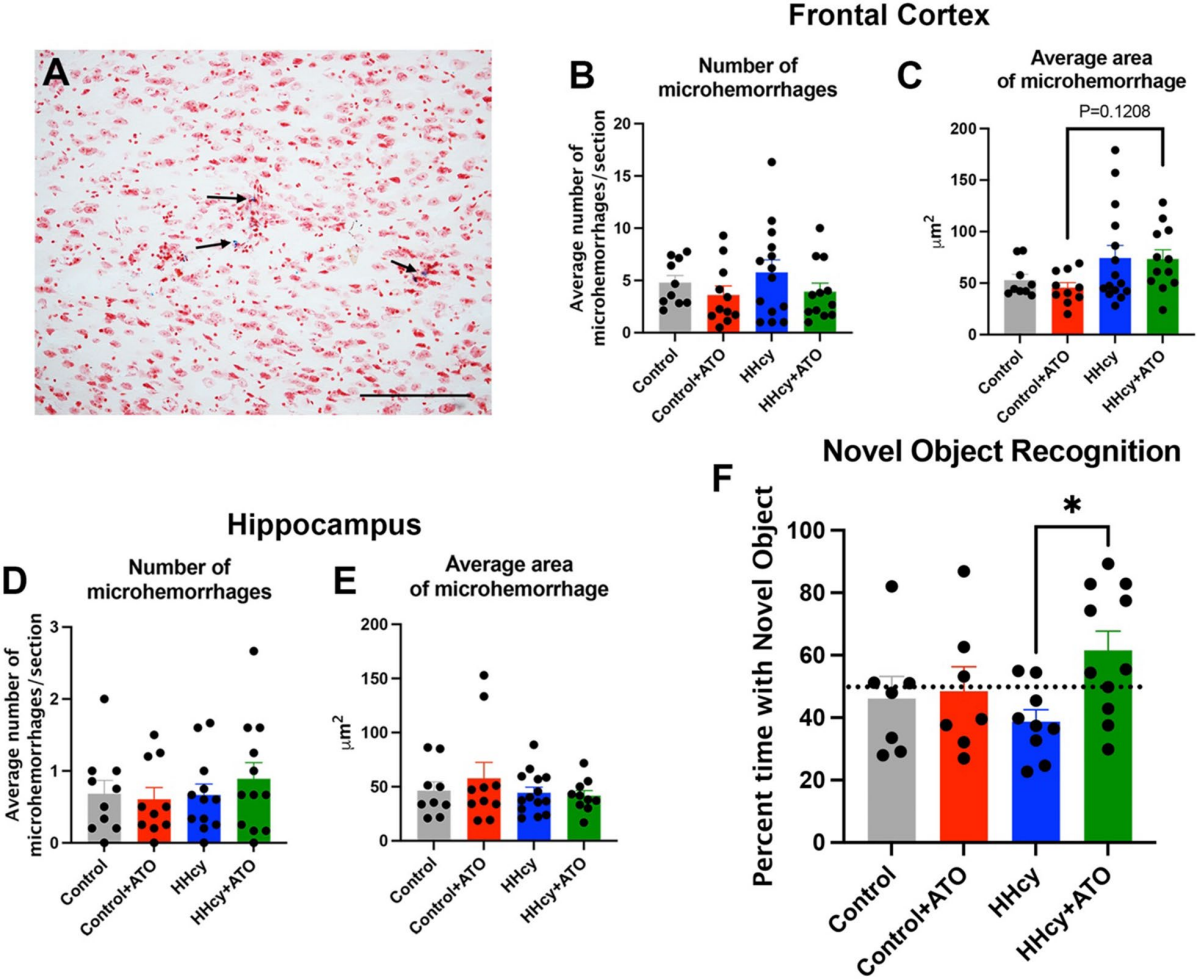
Cognition but not microhemorrhages are improved with atorvastatin treatment. **A)** Representative image of a Prussian blue positive microhemorrhage. Black arrows indicate a Prussian blue positive bleed. Scale bar = 100 µm. Number of microhemorrhages (**B**) and average area of microhemorrhages (**C**) in the frontal cortex are shown. Number of microhemorrhages (**D**) and average area of microhemorrhages (**E**) in the hippocampus are shown. **F)** The percent time spent with the novel object for the novel object recognition task is shown. * indicates P < 0.05

We also wanted to determine if atorvastatin could prevent microhemorrhages induced by the HHcy diet, so we investigated changes in the number and size of microhemorrhages in frontal cortex and hippocampus. The HHcy with atorvastatin mice were trending towards fewer microhemorrhages compared to the HHcy mice but only in the frontal cortex (Fig. 5A–E). However, the average area of the microhemorrhages were trending larger in the HHcy with atorvastatin mice compared to the control with atorvastatin in the frontal cortex (Fig. 5C). There were no significant changes in microhemorrhage number or area in the hippocampus (Fig. 5D, E).

Finally, we ascertained changes in cognition using the novel object recognition task. HHcy with atorvastatin mice spent significantly more time with the novel object compared to HHcy mice (Fig. 5F), suggesting atorvastatin improved cognition.

## Discussion

Considering the population of people 60 years and older is expected to double by 2050 and, with it, the number of dementia cases will triple, it is crucial to identify ways to treat and reduce the risk of dementia [21]. Because statins are already an approved FDA drug, and with epidemiological evidence suggesting that statins reduce the risk of dementia, repurposing statins for treatment and prevention of dementia is a more rapid path for therapeutics than gaining full approval for new drugs. However, pre-clinical studies investigating the mechanisms and types of dementia statins may benefit are still needed. In this study, we used atorvastatin to treat HHcy-induced VCID in mice. Atorvastatin did not reduce plasma total cholesterol levels, not an unexpected finding since none of the mice had elevated plasma cholesterol, it did significantly reduce plasma total homocysteine levels and rescued gene expression changes induced by HHcy. While atorvastatin didn’t impact astrocyte end feet, microglia, and slightly increased the size of microhemorrhages in the frontal cortex, it ultimately rescued cognitive deficits.

In mice, the majority of cholesterol is in the high density lipoprotein (HDL) form due to the lack of cholesteryl ester transfer protein that shuttles cholesteryl esters from HDL to other lipoproteins like low density lipoprotein (LDL) or very low density lipoprotein (VLDL) [22]. Since atorvastatin mainly lowers LDL levels, it has been shown to not change total cholesterol levels in mice [23–25], which aligns with what we have shown in our study. Although we did not have any total cholesterol changes due to atorvastatin in our study, there was a diet effect on cholesterol, with HHcy causing a significant decrease in total cholesterol levels. Animal studies have shown that Hcy increases cholesterol biosynthesis and secretion but decreases protein synthesis of apolipoprotein A1, the main apolipoprotein of HDL, thus decreasing HDL levels [26, 27]. Human studies show similar findings, where HHcy is associated with increased LDL and triglycerides but negatively associated with HDL [28, 29]. Since the majority of cholesterol is in the HDL form in mice, this may explain why the HHcy diet caused a decreased in total cholesterol in our study.

While atorvastatin did not alter cholesterol levels in our study, it did significantly reduce tHcy in the HHcy diet mice, although levels were still in range of moderate levels of HHcy (30–100 µmol/L tHcy) in the HHcy with atorvastatin group. This aligns with the patient literature, which has shown that statins reduce tHcy in plasma. In women with polycystic ovarian syndrome, treatment with atorvastatin or simvastatin led to decreased serum tHcy levels and in another study, patients with hypercholesterolemia who were treated with simvastatin had decreased plasma tHcy [30, 31]. Statins have also been shown to reduce tHcy in patients with the methylenetetrahydrofolate reductase C677T polymorphism that induces HHcy [32]. We also investigated related metabolites to tHcy and found that atorvastatin caused a trending increase in the SAM/SAH ratio compared to HHcy mice. This is a critical finding since SAM is a global methyl donor important for DNA methylation and SAH is an inhibitor of SAM [33]. The SAM/SAH ratio is an indicator of DNA methylation potential, with a lower ratio indicating less DNA methylation potential, suggesting altered methylation patterns and gene expression changes. A change in the SAM/SAH ratio in the HHcy with atorvastatin group could indicate gene expression changes.

Our previous work has shown that HHcy significantly alters gene expression in a glial dependent manner, so we were interested in the glial response to atorvastatin treatment of HHcy [34]. Although NanoString gene expression profiling of glial cells didn’t yield any changes that passed FDR correction, some interesting trends were apparent. The majority of gene changes with a significant P value occurred between control and HHcy mice (35 genes) and between HHcy and HHcy with atorvastatin mice (108 genes). Interestingly, the HHcy diet caused reduced gene expression of every significant gene, similar to what we have previously reported in human brain tissue with HHcy, while atorvastatin increased gene expression [35]. A possible explanation for this could be due to the SAM/SAH ratio and the restored DNA methylation potential. When broken down by cell type, oligodendrocytes had the most gene expression changes associated with atorvastatin, followed by microglia. HHcy has been shown to have a dose response association with white matter lesions and is associated with increased white matter hyperintensity volume, so the majority of gene changes being associated with oligodendrocytes is not surprising [36, 37]. When we put the list of 35 significant genes between control vs HHcy into STRING-db, we saw that one of the nodes centered around several oligodendrocyte markers (*MAG, MBP, OLIG1, GFAP, PLP1, SOX10*) that would again suggest significant oligodendrocyte changes due to HHcy (Additional file 1: Fig. S1). The other node centered around *IL1β* and its related inflammatory markers and microglia were the second glial cell type with the most gene expression changes which aligns with the anti-inflammatory effects of atorvastatin and the pro-inflammatory effects of the HHcy diet [10, 11, 38]. Although when we look at the morphology of the microglia, the trends towards reduced area, process endings and process length in the frontal cortex due to atorvastatin treatment in the HHcy mice compared to controls with atorvastatin would suggest that the microglia are not homeostatic. Further analysis of activated vs non activated microglia with a larger N for staining is required to determine the effect of atorvastatin with HHcy on microglial morphology.

Additional analysis into the NanoString genes using STRING-db allowed us to look at connected genes for the significant genes in the HHcy vs HHcy + ATO group. Similar to the control vs HHcy group, one node centered around oligodendrocyte genes (*OLIG1, MBP, MAG, PLP1, SOX10, S100b, GFAP, AQP4, DMD, SOX2*) (Additional file 1: Fig. S2). However, unlike control vs HHcy, atorvastatin increased expression of these genes, suggesting rescue of these oligodendrocyte changes due to the HHcy diet. Another major node had several genes related to intracellular signaling (*MAPK14, JAK2, STAT2, AKT3, KRAS, PAK1*). Increases in MAPK signaling have been shown to occur due to atorvastatin treatment, although this was not seen in our control vs control + ATO group [39], suggesting this is due to combined HHcy with atorvastatin. With the widespread effects of MAPK signaling, it is difficult to determine the effects of increased MAPK signaling due to atorvastatin treatment, but it presents several different possible avenues for future mechanisms of atorvastatin. Finally, similarly to the control vs HHcy NanoString results, the HHcy vs HHcy + ATO had a node with several inflammatory related genes. Atorvastatin rescued the gene expression decreases in *MSR1 and FCGR1* that were seen in control vs HHcy; however, atorvastatin with HHcy increased complement genes (*C1qc, C1qa*) which was not seen in control vs HHcy. This was surprising since previous data shows that statins either had no effect on complement or attenuated the inflammatory response after complement activation [40, 41]. Further study into the effects of atorvastatin with HHcy into the complement cascade would be necessary to determine if these effects extend to the protein level and if they are beneficial or not.

The majority of epidemiologic data suggests statins reduce the risk of dementia, so we were interested if atorvastatin can prevent cognitive changes induced by the HHcy diet. Using the novel object recognition task, mice on the HHcy diet treated with atorvastatin spent more time with the novel object compared to the HHcy diet mice alone, suggesting they had improved cognition due to atorvastatin treatment. We were also interested if this improved cognition was due to changes in vascular integrity, which is known to be degraded due to the HHcy diet [15, 16, 38]. There were no significant changes in astrocyte end-feet, either due to the HHcy diet or the HHcy diet with atorvastatin, but our N for immunohistochemistry was low, so further studies are required to be sure of this conclusion. There were slight changes in the number of microhemorrhages, with small increases due to HHcy alone (with large variability) and a small reduction in the HHcy mice with atorvastatin, although nothing was significant. However, the area of microhemorrhages were trending larger in the frontal cortex in the HHcy mice with atorvastatin compared to the control mice with atorvastatin. Evidence regarding the association of statins and microbleeds is still unclear, with some studies suggesting no association and others suggesting statins increase microbleeds in a region-specific manner [42, 43]. In our study, the combination of HHcy and atorvastatin trending towards region specific increases in the area of microhemorrhages suggests a role for HHcy in the role of statin associated microhemorrhages. However, the lack of vessel integrity changes suggest that atorvastatin improved cognition through another mechanism outside improving the vasculature or even microglial changes.

## Conclusions

While there was a dietary effect of HHcy, atorvastatin did not change total cholesterol levels, suggesting that any effects seen are due to the pleiotropic effects of atorvastatin. Overall, treatment of HHcy with atorvastatin led to decreased plasma tHcy, increased glial gene expression levels, no changes in astrocyte end-feet or microglial changes, and minimal changes in microhemorrhages, yet led to improved cognition. The lack of vascular changes suggest that the reduced tHcy and the rescued gene expression changes could be the potential mechanism behind the improved cognition in the HHcy mice treated with atorvastatin. While improved cognition is a promising outcome, further studies are required to understand the mechanism behind this improvement and whether statins are a potential therapeutic treatment for HHcy patients who do not respond to B vitamin supplements.

### Supplementary Information

**Additional file 1: Table S1.** List of significant genes for control vs control + ATO. **Figure S1.** STRING interaction network based off significant genes for control vs HHcy. The list of significant genes with the fold change were put into STRING-db.org to determine connections. A high confidence (0.7) interaction score was applied and disconnected genes were excluded. The depth of color of the halo around each gene is respective of that genes fold change. **Table S2.** List of significant genes for control vs HHcy. **Table S3.** List of significant genes for control vs HHcy + ATO. **Figure S2.** STRING interaction network based off significant genes for HHcy vs HHcy + ATO. The list of significant genes with the fold change were put into STRING-db.org to determine connections. A high confidence (0.7) interaction score was applied and disconnected genes were excluded. The depth of color of the halo around each gene is respective of that genes fold change. **Table S4.** List of significant genes for HHcy vs HHcy + ATO.

## Data Availability

All data generated or analyzed during this study are included in this published article and its supplementary files.

## Abbreviations

AD: Alzheimer’s disease
AQP4: Aquaporin 4
DP71: Dystrophin 71
ERBB3: Receptor tyrosine-protein kinase erbB-3
FDR: False discovery rate
HDL: High density lipoprotein
HHcy: Hyperhomocysteinemia
IBA-1: Ionized calcium binding adaptor molecule 1
LDL: Low density lipoprotein
MAG: Myelin associated glycoprotein
OLIG1: Oligodendrocyte transcription factor 1
OLFM3: Olfactomedin 3
PLP1: Proteolipid 1
SAH: S-adenosyl homocysteine
SAM: S-adenosyl methionine
tHcy: Total homocysteine
VCID: Vascular contributions to cognitive impairment and dementia
VLDL: Very low density lipoprotein
