# Supplementary material for: Atorvastatin rescues hyperhomocysteinemia-induced cognitive deficits and neuroinflammatory gene changes
Source: J Neuroinflammation. 2023 Sep 1;20:199. doi: 10.1186/s12974-023-02883-x (PMC10474691; doi:10.1186/s12974-023-02883-x)
Supplement: Supplementary file 1 — Additional file 1: Table S1. List of significant genes for control vs control + ATO. Figure S1. STRING interaction network based off significant genes for control vs HHcy. The list of significant genes with the fold change were put into STRING-db.org to determine connections. A high confidence (0.7) interaction score was applied and disconnected genes were excluded. The depth of color of the halo around each gene is respective of that genes fold change. Table S2. List of significant genes for control vs HHcy. Table S3. List of significant genes for control vs HHcy + ATO. Figure S2. STRING interaction network based off significant genes for HHcy vs HHcy + ATO. The list of significant genes with the fold change were put into STRING-db.org to determine connections. A high confidence (0.7) interaction score was applied and disconnected genes were excluded. The depth of color of the halo around each gene is respective of that genes fold change. Table S4. List of significant genes for HHcy vs HHcy + ATO. [file 12974_2023_2883_MOESM1_ESM.docx]

**Table S1: List of significant genes for control vs control+ATO**

| **Gene** | **Effect Size** | **P-value** | **Q-value** |
| --- | --- | --- | --- |
| ***Cd200r1*** | -0.367849345 | 0.029204921 | 0.8935803 |
| ***Dlx2*** | 0.368174078 | 0.018990554 | 0.8935803 |
| ***Erbb3*** | -0.243271814 | 0.023138454 | 0.8935803 |
| ***Fa2h*** | -0.223320196 | 0.012788385 | 0.8935803 |
| ***Fcrlb*** | 0.435467102 | 0.040447191 | 0.8935803 |
| ***Gpnmb*** | -0.337612335 | 0.044559846 | 0.8935803 |


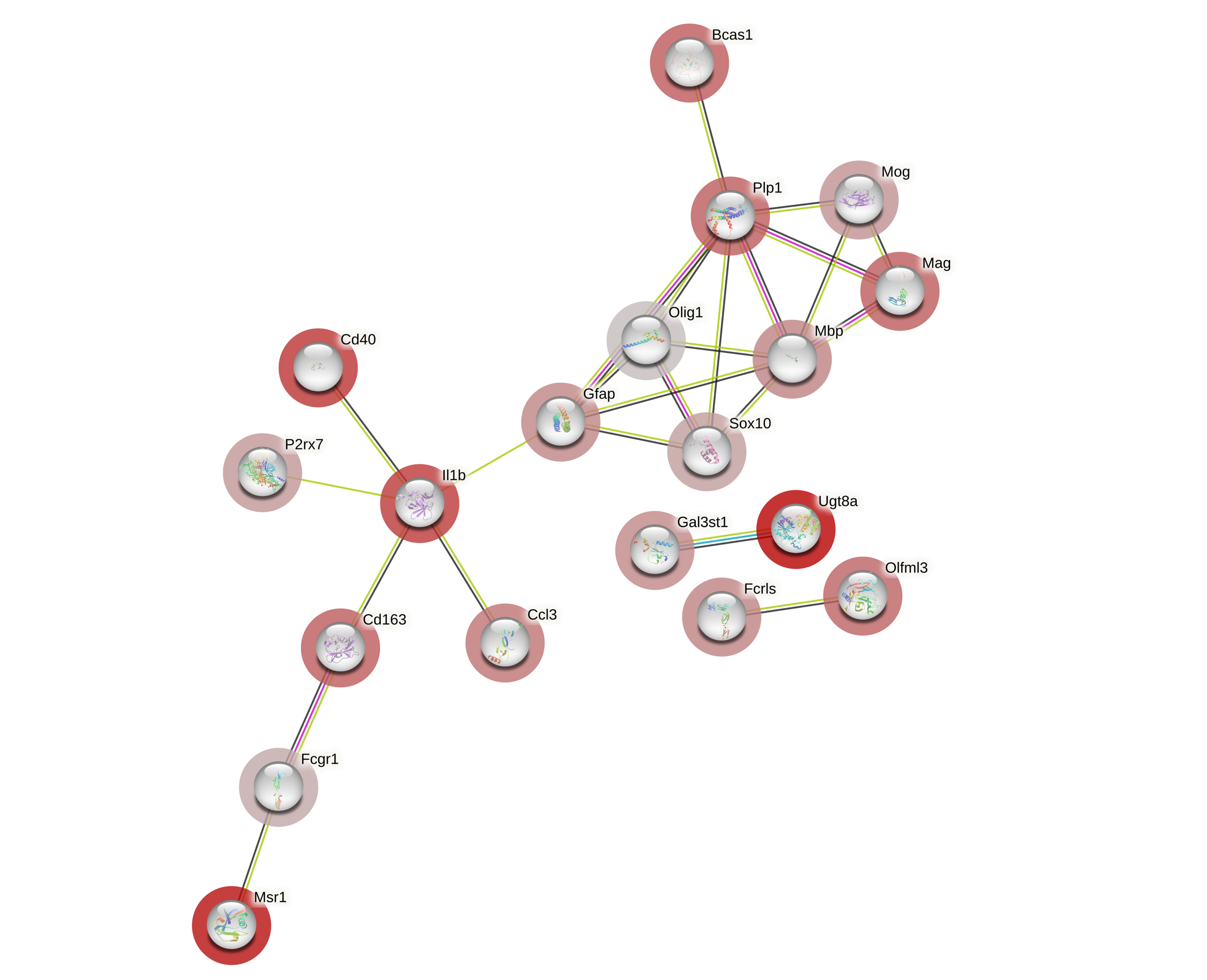


**Figure S1: STRING interaction network based off significant genes for control vs HHcy.** The list of significant genes with the fold change were put into STRING-db.org to determine connections. A high confidence (0.7) interaction score was applied and disconnected genes were excluded. The depth of color of the halo around each gene is respective of that genes fold change.

**Table S2: List of significant genes for control vs HHcy**

| **Gene** | **Effect Size** | **P-value** | **Q-value** |
| --- | --- | --- | --- |
| ***Aldh1a1*** | -0.236553235 | 0.045141494 | 0.999690743 |
| ***Bcas1*** | -0.322441673 | 0.011430636 | 0.563239003 |
| ***Ccl3*** | -0.283443609 | 0.031446257 | 0.917336625 |
| ***Cd163*** | -0.319220447 | 0.00877432 | 0.550521444 |
| ***Cd40*** | -0.378368666 | 0.00535025 | 0.466660688 |
| ***Cfi*** | -0.410463453 | 0.041702174 | 0.992006267 |
| ***Cspg4*** | -0.226829001 | 0.036958349 | 0.992006267 |
| ***Erbb3*** | -0.323672685 | 0.003165941 | 0.466660688 |
| ***Ermn*** | -0.308725806 | 0.014408934 | 0.615710589 |
| ***Fa2h*** | -0.295753943 | 0.000662 | 0.259835144 |
| ***Fcgr1*** | -0.209107167 | 0.044477143 | 0.999690743 |
| ***Fcrls*** | -0.260690403 | 0.008602182 | 0.550521444 |
| ***Gal3st1*** | -0.254991697 | 0.0151451 | 0.615710589 |
| ***Gfap*** | -0.25984508 | 0.011480031 | 0.563239003 |
| ***Gjb1*** | -0.290534572 | 0.008615565 | 0.550521444 |
| ***Gpnmb*** | -0.339311642 | 0.022750019 | 0.804614924 |
| ***Il1b*** | -0.36918066 | 0.040950474 | 0.992006267 |
| ***Klk6*** | -0.364461905 | 0.024599692 | 0.804614924 |
| ***Lair1*** | -0.319055858 | 0.020364519 | 0.761245099 |
| ***Mag*** | -0.32034835 | 0.000606321 | 0.259835144 |
| ***Mbp*** | -0.260802252 | 0.012924699 | 0.596816972 |
| ***Mog*** | -0.240007511 | 0.004411719 | 0.466660688 |
| ***Msr1*** | -0.428780547 | 0.031059361 | 0.917336625 |
| ***Ninj2*** | -0.29546946 | 0.009116916 | 0.550521444 |
| ***Olfml3*** | -0.31101886 | 0.011216083 | 0.563239003 |
| ***Olig1*** | -0.179013158 | 0.035758717 | 0.992006267 |
| ***P2rx7*** | -0.23217535 | 0.005212628 | 0.466660688 |
| ***Plekhb1*** | -0.205515155 | 0.031551706 | 0.917336625 |
| ***Plp1*** | -0.319428815 | 0.003788017 | 0.466660688 |
| ***Plxnb3*** | -0.283768458 | 0.023788284 | 0.804614924 |
| ***Sirt5*** | -0.24930139 | 0.003548673 | 0.466660688 |
| ***Slc2a5*** | -0.180442236 | 0.037944808 | 0.992006267 |
| ***Sox10*** | -0.224133811 | 0.015686894 | 0.615710589 |
| ***Ugt8a*** | -0.45093051 | 0.001765381 | 0.461941253 |
| ***Unc79*** | -0.16866175 | 0.041691004 | 0.992006267 |

**Table S3: List of significant genes for control vs HHcy+ATO**

| **Gene** | **Effect Size** | **P-value** | **Q-value** |
| --- | --- | --- | --- |
| ***Cd74*** | 0.453489138 | 0.03843104 | 0.99530208 |
| ***Erbb3*** | -0.263263408 | 0.008740059 | 0.99530208 |
| ***Gpnmb*** | -0.303016382 | 0.043138341 | 0.99530208 |
| ***Hpgds*** | -0.232013303 | 0.041488402 | 0.99530208 |
| ***Hspa1a/b*** | -0.370422246 | 0.021880356 | 0.99530208 |
| ***Il1b*** | -0.370912511 | 0.036432357 | 0.99530208 |
| ***Myct1*** | -0.342985072 | 0.002745621 | 0.99530208 |
| ***Tgm1*** | 0.534771411 | 0.020771565 | 0.99530208 |
| ***Thrb*** | 0.200973407 | 0.02031553 | 0.99530208 |


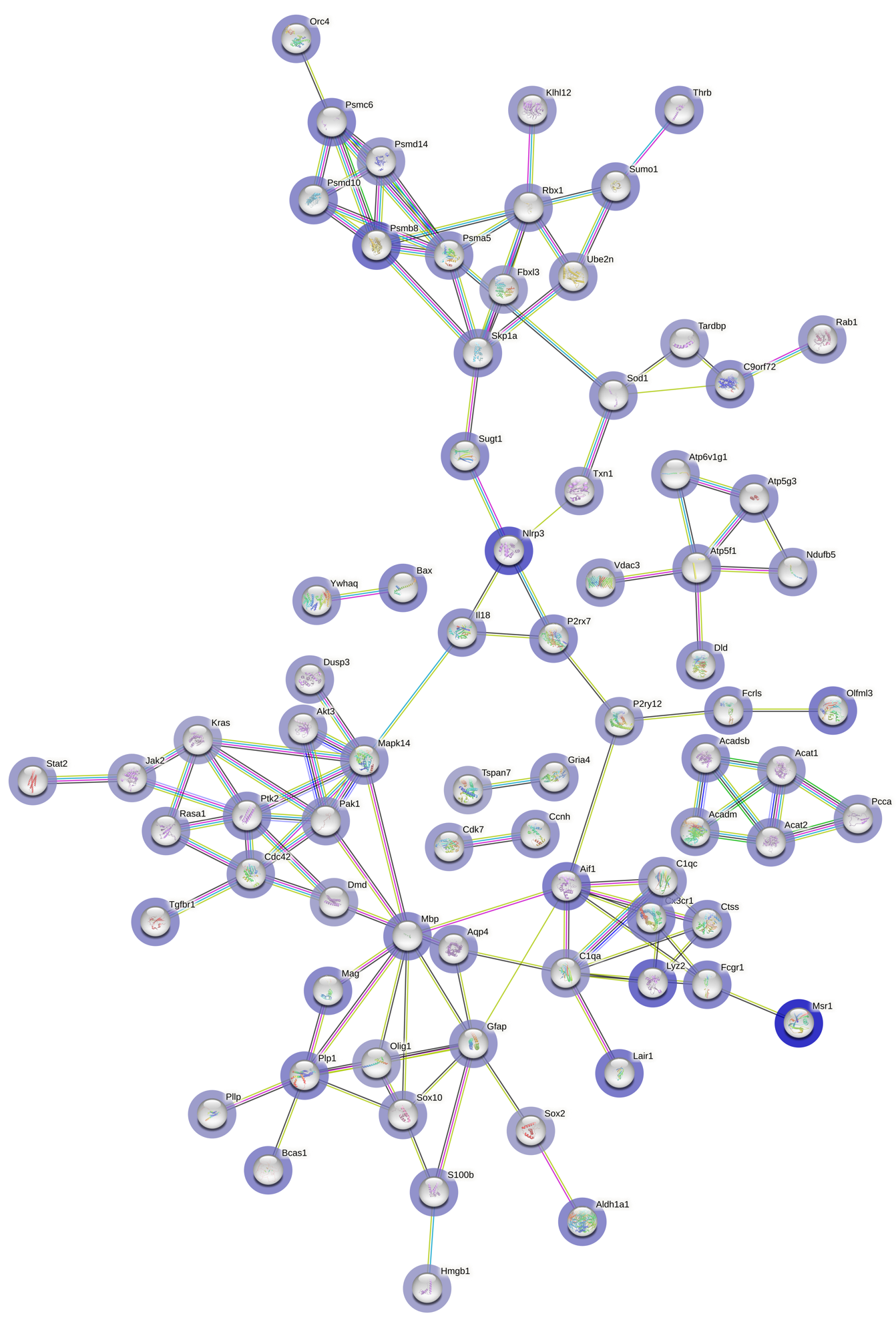


**Figure S2:** **STRING interaction network based off significant genes for HHcy vs HHcy+ATO.** The list of significant genes with the fold change were put into STRING-db.org to determine connections. A high confidence (0.7) interaction score was applied and disconnected genes were excluded. The depth of color of the halo around each gene is respective of that genes fold change.

**Table 4: List of significant genes for HHcy vs HHcy+ATO**

| **Gene** | **Effect Size** | **P-value** | **Q-value** |
| --- | --- | --- | --- |
| ***Acadm*** | 0.180888617 | 0.016536389 | 0.323709865 |
| ***Acadsb*** | 0.202471225 | 0.025303541 | 0.323709865 |
| ***Acat1*** | 0.176297036 | 0.025985641 | 0.323709865 |
| ***Acat2*** | 0.195073049 | 0.046915773 | 0.346998103 |
| ***Adora2b*** | 0.237475058 | 0.034969733 | 0.323709865 |
| ***Ahcyl1*** | 0.130255657 | 0.040148717 | 0.323709865 |
| ***Aif1*** | 0.269348892 | 0.010414377 | 0.323709865 |
| ***Akt3*** | 0.166889688 | 0.012887389 | 0.323709865 |
| ***Aldh1a1*** | 0.236068675 | 0.008843609 | 0.323709865 |
| ***Aqp4*** | 0.194360663 | 0.017807892 | 0.323709865 |
| ***Arl6ip5*** | 0.183664732 | 0.047297831 | 0.346998103 |
| ***Arpc5l*** | 0.192106466 | 0.040382148 | 0.323709865 |
| ***Atg5*** | 0.149327947 | 0.036103517 | 0.323709865 |
| ***Atp5g3*** | 0.190783105 | 0.021711332 | 0.323709865 |
| ***Atp5pb*** | 0.19402139 | 0.021149014 | 0.323709865 |
| ***Atp6v1g1*** | 0.186422446 | 0.026799479 | 0.323709865 |
| ***Bax*** | 0.22130965 | 0.000534625 | 0.323709865 |
| ***Bcas1*** | 0.232914654 | 0.022896953 | 0.323709865 |
| ***C1qa*** | 0.163704478 | 0.031394608 | 0.323709865 |
| ***C1qc*** | 0.183342959 | 0.022124758 | 0.323709865 |
| ***C9orf72*** | 0.208075051 | 0.003751181 | 0.323709865 |
| ***Calm2*** | 0.174236504 | 0.044414479 | 0.335243905 |
| ***Ccdc127*** | 0.16688657 | 0.019355668 | 0.323709865 |
| ***Ccnh*** | 0.181759695 | 0.039247648 | 0.323709865 |
| ***Cdc42*** | 0.182197594 | 0.035014613 | 0.323709865 |
| ***Cdk7*** | 0.166961272 | 0.033525732 | 0.323709865 |
| ***Chchd3*** | 0.190004167 | 0.024226568 | 0.323709865 |
| ***Ctss*** | 0.208743877 | 0.031323295 | 0.323709865 |
| ***Cx3cr1*** | 0.222348417 | 0.006494335 | 0.323709865 |
| ***Ddah1*** | 0.178206517 | 0.008630393 | 0.323709865 |
| ***Dld*** | 0.196734027 | 0.038076204 | 0.323709865 |
| ***Dmd*** | 0.144055209 | 0.017756125 | 0.323709865 |
| ***Dusp3*** | 0.170315726 | 0.009956411 | 0.323709865 |
| ***Epb41l2*** | 0.129747337 | 0.026183577 | 0.323709865 |
| ***Ermn*** | 0.213866839 | 0.02496065 | 0.323709865 |
| ***Fa2h*** | 0.168270599 | 0.028004004 | 0.323709865 |
| ***Fabp5*** | 0.215693888 | 0.002521326 | 0.323709865 |
| ***Fbxl3*** | 0.148833566 | 0.044072676 | 0.335243905 |
| ***Fcgr1*** | 0.242648176 | 0.037519837 | 0.323709865 |
| ***Fcrls*** | 0.213631422 | 0.007042213 | 0.323709865 |
| ***Gfap*** | 0.183631302 | 0.039689443 | 0.323709865 |
| ***Gmpr2*** | 0.183301873 | 0.035483997 | 0.323709865 |
| ***Gria4*** | 0.18081404 | 0.044225042 | 0.335243905 |
| ***Gusb*** | 0.198823364 | 0.022684891 | 0.323709865 |
| ***Hexb*** | 0.172196436 | 0.040104705 | 0.323709865 |
| ***Hmgb1*** | 0.151209589 | 0.042644252 | 0.335243905 |
| ***Icam2*** | 0.246348829 | 0.003375068 | 0.323709865 |
| ***Il18*** | 0.199756636 | 0.034636726 | 0.323709865 |
| ***Jak2*** | 0.15619897 | 0.02525301 | 0.323709865 |
| ***Klhl12*** | 0.171664705 | 0.032514356 | 0.323709865 |
| ***Kras*** | 0.140622772 | 0.040412187 | 0.323709865 |
| ***Lair1*** | 0.285915414 | 0.006306559 | 0.323709865 |
| ***Lamtor3*** | 0.164536782 | 0.037845718 | 0.323709865 |
| ***Lyz2*** | 0.334262896 | 0.017470311 | 0.323709865 |
| ***Mag*** | 0.228769758 | 0.005301663 | 0.323709865 |
| ***Mapk14*** | 0.180842828 | 0.020819161 | 0.323709865 |
| ***Mbp*** | 0.221284017 | 0.012310402 | 0.323709865 |
| ***Msr1*** | 0.534372925 | 0.017194984 | 0.323709865 |
| ***Ndufb5*** | 0.178822355 | 0.016872759 | 0.323709865 |
| ***Nlrp3*** | 0.375599189 | 0.017921508 | 0.323709865 |
| ***Olfml3*** | 0.271567843 | 0.010167299 | 0.323709865 |
| ***Olig1*** | 0.145677785 | 0.027882663 | 0.323709865 |
| ***Orc4*** | 0.204720859 | 0.013387153 | 0.323709865 |
| ***P2rx7*** | 0.211316829 | 0.005676554 | 0.323709865 |
| ***P2ry12*** | 0.173300063 | 0.022025772 | 0.323709865 |
| ***Pak1*** | 0.173736221 | 0.033803203 | 0.323709865 |
| ***Pcca*** | 0.174163976 | 0.039231028 | 0.323709865 |
| ***Phyh*** | 0.151747788 | 0.015432008 | 0.323709865 |
| ***Plekhb1*** | 0.170281541 | 0.036246007 | 0.323709865 |
| ***Pllp*** | 0.171254143 | 0.039022848 | 0.323709865 |
| ***Plp1*** | 0.235765615 | 0.016487414 | 0.323709865 |
| ***Psma5*** | 0.197276135 | 0.022449719 | 0.323709865 |
| ***Psmb8*** | 0.3037816 | 0.011979853 | 0.323709865 |
| ***Psmc6*** | 0.239028766 | 0.004377273 | 0.323709865 |
| ***Psmd10*** | 0.225626103 | 0.034733524 | 0.323709865 |
| ***Psmd14*** | 0.166452047 | 0.03797684 | 0.323709865 |
| ***Ptk2*** | 0.1935595 | 0.012178937 | 0.323709865 |
| ***Rab1a*** | 0.171289968 | 0.035064329 | 0.323709865 |
| ***Rasa1*** | 0.179382451 | 0.027570517 | 0.323709865 |
| ***Rbx1*** | 0.18763675 | 0.015579979 | 0.323709865 |
| ***S100b*** | 0.206997054 | 0.035107696 | 0.323709865 |
| ***Scoc*** | 0.174913202 | 0.024172869 | 0.323709865 |
| ***Selenos*** | 0.200224222 | 0.031492876 | 0.323709865 |
| ***Sirt5*** | 0.20204306 | 0.002760462 | 0.323709865 |
| ***Skp1a*** | 0.205077372 | 0.015900004 | 0.323709865 |
| ***Slc8a1*** | 0.159056904 | 0.005249714 | 0.323709865 |
| ***Sod1*** | 0.181749062 | 0.023519513 | 0.323709865 |
| ***Sox10*** | 0.177688406 | 0.033884554 | 0.323709865 |
| ***Sox2*** | 0.145654602 | 0.028776197 | 0.323709865 |
| ***Stambpl1*** | 0.161177153 | 0.044961306 | 0.336139285 |
| ***Stat2*** | 0.167180524 | 0.021468704 | 0.323709865 |
| ***Sugt1*** | 0.219065133 | 0.006379909 | 0.323709865 |
| ***Sumo1*** | 0.208824808 | 0.011603433 | 0.323709865 |
| ***Tardbp*** | 0.158836556 | 0.043936803 | 0.335243905 |
| ***Tgfbr1*** | 0.212229402 | 0.02102113 | 0.323709865 |
| ***Thrb*** | 0.20491962 | 0.006194132 | 0.323709865 |
| ***Tie1*** | -0.191017409 | 0.029832091 | 0.323709865 |
| ***Trac*** | 0.182185714 | 0.024901563 | 0.323709865 |
| ***Trim37*** | 0.156219742 | 0.037375604 | 0.323709865 |
| ***Tspan7*** | 0.128189556 | 0.042781185 | 0.335243905 |
| ***Txn1*** | 0.176183576 | 0.038488956 | 0.323709865 |
| ***Ube2n*** | 0.17489752 | 0.048362012 | 0.348955772 |
| ***Ugt8a*** | 0.315742198 | 0.00956944 | 0.323709865 |
| ***Unc79*** | 0.233651885 | 0.004652824 | 0.323709865 |
| ***Vdac3*** | 0.175549295 | 0.038747903 | 0.323709865 |
| ***vegfb*** | 0.178416601 | 0.011693821 | 0.323709865 |
| ***Ywhaq*** | 0.180155481 | 0.020677052 | 0.323709865 |
| ***Zfp365*** | 0.149442002 | 0.034038215 | 0.323709865 |
